# Atherogenic Lipoprotein Determinants of Cardiovascular Disease and Residual Risk Among Individuals With Low Low‐Density Lipoprotein Cholesterol

**DOI:** 10.1161/JAHA.117.005549

**Published:** 2017-07-21

**Authors:** Patrick R. Lawler, Akintunde O. Akinkuolie, Audrey Y. Chu, Svati H. Shah, William E. Kraus, Damian Craig, Latha Padmanabhan, Robert J. Glynn, Paul M Ridker, Daniel I. Chasman, Samia Mora

**Affiliations:** ^1^ Center for Lipid Metabolomics Brigham and Women's Hospital Harvard Medical School Boston MA; ^2^ Cardiovascular Division Brigham and Women's Hospital Harvard Medical School Boston MA; ^3^ Preventive Medicine Division Brigham and Women's Hospital Harvard Medical School Boston MA; ^4^ Peter Munk Cardiac Centre University Health Network Toronto Ontario Canada; ^5^ Heart and Stroke Richard Lewar Centre of Excellence in Cardiovascular Research University of Toronto Ontario Canada; ^6^ Division of Cardiology and Duke Molecular Physiology Institute Duke University School of Medicine Durham NC; ^7^ Harvard T. H. Chan School of Public Health Boston MA

**Keywords:** atherosclerosis, lipids and lipoproteins, metabolomics, nuclear magnetic resonance spectroscopy, prevention, Cardiovascular Disease, Lipids and Cholesterol, Primary Prevention, Atherosclerosis

## Abstract

**Background:**

Levels of LDL (low‐density lipoprotein) cholesterol in the population are declining, and increasing attention is being focused on residual lipid‐related pathways of atherosclerotic cardiovascular disease risk beyond LDL cholesterol. Among individuals with low (<130 mg/dL) LDL cholesterol, we undertook detailed profiling of circulating atherogenic lipoproteins in relation to incident cardiovascular disease in 2 populations.

**Methods and Results:**

We performed proton nuclear magnetic resonance spectroscopy to quantify concentrations of LDL and VLDL (very low‐density lipoprotein) particle subclasses in 11 984 JUPITER trial participants (NCT00239681). Adjusted Cox models examined cardiovascular disease risk associated with lipoprotein measures according to treatment allocation. Risk (adjusted hazard ratio [95%CI] per SD increment) among placebo‐allocated participants was associated with total LDL particles (1.19 [1.02, 1.38]) and total VLDL particles (1.21 [1.04, 1.41]), as well as apolipoprotein B, non–high‐density lipoprotein cholesterol, and triglycerides, but not LDL‐c. Rosuvastatin reduced LDL measures but had variable effects on triglyceride and VLDL measures. On‐statin levels of the smallest VLDL particle subclass were associated with a 68% per‐SD (adjusted hazard ratio 1.68 [1.28, 2.22]) increase in residual risk—this risk was related to VLDL cholesterol and not triglyceride or larger VLDL particles. There was evidence that residual risk prediction during statin therapy could be significantly improved through the inclusion of key VLDL measures (Harrell C‐index 0.780 versus 0.712; *P*<0.0001). In an independent, prospective cohort of 4721 individuals referred for cardiac catheterization (CATHGEN), similar patterns of lipoprotein‐related risk were observed.

**Conclusions:**

Atherogenic lipoprotein particle concentrations were associated with cardiovascular disease risk when LDL cholesterol was low. VLDL lipoproteins, particularly the smallest remnant subclass, may represent unused targets for risk prediction and potential therapeutic intervention for reducing residual risk.

**Clinical Trial Registration:**

URL: http://www.clinicaltrials.gov. Unique identifier: NCT00239681.

**Clinical Perspective**

**What Is New?**

- Among a population intended to represent the growing number of individuals with low low‐density lipoprotein cholesterol, atherogenic lipoprotein particle concentrations (low‐density lipoprotein and very low‐density lipoprotein particles) were markers of residual atherosclerotic cardiovascular disease risk.
- Among individuals with the lowest low‐density lipoprotein cholesterol on statin, the smallest subclass of very low‐density lipoprotein was strongly associated with residual risk.
- This risk was related to the cholesterol carried in small very low‐density lipoprotein, and not triglyceride.
- Similar patterns were observed in an independent, diverse population of individuals referred for cardiac catheterization at a single center.

**What Are the Clinical Implications?**

- Species of triglyceride‐rich lipoproteins (very low‐density lipoproteins) may represent unused clinical targets for risk prediction and potential therapeutic intervention in the prevention of atherosclerotic cardiovascular disease, particularly among individuals with low‐density lipoprotein cholesterol reduction on statin.
- Triglyceride level may incompletely reflect risk differentially related to triglyceride‐rich lipoprotein subclasses.
- Detailed phenotyping of the circulating lipid and lipoprotein milieu could improve risk assessment, as well as advance the evaluation of emerging therapies to prevent cardiovascular disease.

## Introduction

Population levels of LDL‐c (low‐density lipoprotein cholesterol) are declining with increasing adherence to healthy lifestyle and pharmacologic interventions.^1^, ^2^ However, cardiovascular disease (CVD) events remain prevalent among individuals with low or normal LDL‐c, both pretreatment and during high‐intensity statin therapy,^3^ a phenomenon referred to as residual risk. In parallel, as population levels of LDL‐c have declined over recent years, studies have demonstrated time‐dependent changes in the composition and morphology of human atherosclerotic plaque,^4^ suggesting shifting mechanisms of atherogenesis and plaque disruption amid this changing risk factor exposure and changing epidemiology of CVD.^5^ Numerous studies have suggested that LDL‐c does not account for all of the risk conferred by atherogenic plasma lipids and the insulating lipid and protein assemblies that transport them in the bloodstream, lipoproteins,^6^, ^7^ and thus, it is possible that residual lipid and lipoprotein risk pathways or markers that were previously eclipsed in significance by LDL‐c are likely to emerge as increasingly important determinants of CVD.

**Figure 1:**
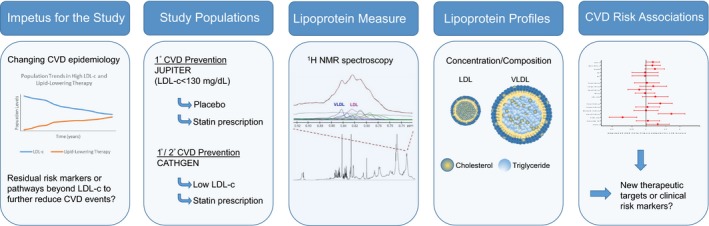
Overview of the study: impetus for the study, study populations, lipoprotein measures, lipoprotein profiling, and results. CATHGEN indicates CATHeterization GENetics biorepository; CVD, cardiovascular disease; JUPITER, Justification for the Use of Statins in Prevention: an Intervention Trial Evaluating Rosuvastatin; LDL, low‐density lipoproteins; LDL‐c, low‐density lipoprotein cholesterol; NMR, nuclear magnetic resonance spectroscopy; VLDL, very low‐density lipoproteins.

To understand the residual markers of lipid‐ and lipoprotein‐related risk in the era of LDL‐c reduction and to forecast the emergence of such alternate risk pathways, we examined populations of individuals with low (<130 mg/dL) LDL‐c with advanced lipoprotein profiling. Detailed lipoprotein phenotyping by proton nuclear magnetic resonance (^1^H NMR) spectroscopy can quantify concentrations, subclass distributions, composition, and size of atherogenic LDL particles (LDL‐p) and very low‐density lipoprotein particles (VLDL‐p) in circulation.^8^, ^9^ Accordingly, this study was designed (Figure 1) to (1) identify potential NMR‐measured lipoproteins associated with incident CVD risk in a population of individuals with naturally normal or low LDL‐c, (2) understand the changes in the lipoprotein milieu accompanying high‐intensity statin therapy, and (3) identify lipoprotein subclasses associated with on‐statin risk of CVD events despite achieved low levels of LDL‐c. We sought to extend the findings by examining an independent, diverse cohort of individuals referred for cardiac catheterization (CATHGEN).

## Material and Methods

### Study Design and Population

The primary study population is derived from a primary‐prevention randomized controlled clinical trial of rosuvastatin 20 mg versus placebo (Justification for the Use of Statins in Prevention: an Intervention Trial Evaluating Rosuvastatin, JUPITER; NCT00239681).^10^ The JUPITER trial randomized individuals (women ≥60 years, men ≥50 years) without prior history of CVD or diabetes mellitus, all of whom had LDL‐c <130 mg/dL, high‐sensitivity C‐reactive protein (hsCRP ≥2.0 mg/L), and triglycerides ≤500 mg/dL. The relative risk reduction for the primary incident CVD end point was 44%.^10^

We performed ^1^H NMR lipoprotein measurements on fasting blood samples from 11 984 participants (of whom 9222 also had 1‐year samples) who consented to and provided additional blood collection with sufficient plasma available. In the placebo‐allocated arm, baseline lipoprotein measurements were used to identify lipoproteins associated with risk of incident CVD in untreated individuals. In the statin‐allocated arm, (1) change in lipoprotein measurements from baseline to 12 months after randomization was used to understand the effects of statin therapy on lipoprotein subfractions, and (2) on‐statin (12 month) lipoprotein measurements were used to identify lipoproteins associated with residual on‐statin risk of incident CVD.

### Laboratory Analysis

Standard lipid measurements were performed on fasting samples by a central laboratory.^11^ LDL‐c was calculated by the Friedewald equation (total cholesterol minus HDL‐c minus triglycerides/5) when triglycerides were <400 mg/dL, and measured by ultracentrifugation when triglycerides were ≥400 mg/dL. Triglycerides were measured using a colorimetric assay. Measurement of apolipoproteins B (apoB) was via immunonephelometry by using a Behring nephelometric assay.^12^

After trial completion, ^1^H NMR spectroscopy (400 MHz) LipoProfile III measurements were performed on stored plasma samples by LipoScience, Inc (Raleigh, NC; now LabCorp). NMR was used to quantify total LDL‐p and its lipoprotein subclass concentrations (small and large LDL‐p, and intermediate density lipoprotein [IDL‐p]), and average LDL particle size. Total VLDL‐p and its subclasses (small, medium, and large) were quantified, as well the concentration of VLDL and chylomicron triglyceride (VLDL/CM TG) and cholesterol (VLDL‐c). We also examined formulaic remnant cholesterol, which was calculated as total cholesterol minus HDL‐c minus LDL‐c. (Calculating remnant cholesterol as triglycerides divided by 5^13^ yielded nearly identical results, as expected, based on use of the Friedewald equation.)

### Outcomes

The primary outcome was the trial primary end point, a composite incident CVD end point defined as the occurrence of either myocardial infarction, stroke, hospitalization for unstable angina, arterial revascularization, or cardiovascular death.^10^ An expanded end point of CVD or all‐cause mortality was also examined, consistent with prior JUPITER biomarker analyses.^14^

### Extension to Other Populations

We sought to extend the JUPITER findings in an independent, prospective cohort encompassing a diverse population referred for cardiac catheterization at Duke University from 2001 to 2010 (CATHGEN; dmpi.duke.edu/cathgen).^15^ As in JUPITER, we focused on individuals with LDL‐c <130 mg/dL and TG <500 mg/dL (N=2149 individuals). (The hsCRP was not routinely measured.) We also examined the subset of participants with confirmed statin prescription (N=833) as well as, more broadly, risks among all comers in the cohort without LDL‐c stratification. Lipoprotein subclasses were measured using the same NMR assay as in JUPITER. Fatal or nonfatal myocardial infarction was the primary outcome in this mixed population of individuals both with and without established CVD. Cox models included all covariables used in the JUPITER analyses except for hsCRP, which was not routinely measured.

### Statistical Analyses

Medians (25th, 75th percentiles) were displayed for continuous variables. Lipoprotein change was evaluated using the Wilcoxon signed‐rank test. Exposure time was calculated as the time from randomization to end‐point occurrence or censoring (the latter including censoring at time of noncardiovascular mortality). Cox proportional hazards models were used to calculate hazard ratios and 95% CIs for CVD in relation to per‐SD increments in measures. Nonnormally distributed data were (natural) log transformed. Additionally, risk was examined across lipoprotein tertiles. Models were adjusted for age, sex, race, smoking, family history of premature coronary disease, body‐mass index, systolic blood pressure, fasting glucose, and hsCRP. Sensitivity analyses mutually adjusting for other NMR lipoprotein subclasses were performed,^16^ as well as adjusting for HDL‐c. Additionally, a minority of individuals in these analyses had events before on‐treatment NMR measurement; however, the majority of events occurred after measurement, and in sensitivity analyses beginning the follow‐up at time of NMR measurement, the results were not appreciably different, although with less power some associations were no longer significant. We assessed predictive model performance by assessing the change in Harrell C‐index (and quantitatively with the likelihood ratio test) for 2 hierarchically nested models predicting primary CVD events: (1) a model that incorporated all variables from the Cox model (above) plus standard lipids (LDL‐c, HDL‐c, and triglyceride), and (2) a full model including all variables from model 1 plus all of the atherogenic NMR lipoprotein measurements. Additionally, a more parsimonious model was selected through backwards elimination (retention threshold, *P*<0.10) starting with all variables in model 2; discrimination was assessed with Harrell C‐index, and the likelihood ratio test was used to determine if model 2 provided significantly improved discrimination over this parsimonious model. Analyses were performed using SAS version 9.3 (Cary, NC). A 2‐tailed *P*<0.05 was considered statistically significant. The study was approved by a local Institutional Review Board, and subjects provided informed consent.

## Results

### Baseline Characteristics

Study participants had a median (25th, 75th percentiles) age of 66 (60, 71) years, and were 36% female. Except for race/ethnicity, participants in the current study were generally representative of those in the original JUPITER trial (Table 1).^10^ Median baseline LDL‐c was 109 (95, 120) mg/dL, triglycerides were 119 (87, 169) mg/dL, and hsCRP was 4.10 (2.75, 6.70) mg/L.

**Table 1:** Baseline Characteristics in the Study Sample Vs the Original JUPITER Cohort

Characteristic	Current Study (N=11 984)	JUPITER (N=17 802)	Not in Current Study (N=5818)
	Median (IQR) or N (%)	Median (IQR) or N (%)	Median (IQR) or N (%)
Age	66 (60, 71)	66 (60, 71)	66 (61, 71)
Women	4360 (36)	6801 (38.2)	2411 (42)
Rosuvastatin	5934 (50)	8901 (50.0)	2967 (51)
Race/ethnicity			
White	9894 (82.6)	12 683 (71.3)	2789 (48)
Black	746 (6.2)	2224 (12.5)	1478 (25)
Asian	178 (1.5)	283 (1.6)	105 (1.8)
Hispanic	1076 (9.0)	2261 (12.7)	1185 (20)
Other/unknown	88 (0.7)	349 (2.0)	261 (5)
Body‐mass index, kg/m^2^	28.4 (25.5, 32.0)	28.4 (25.3, 32.0)	28.0 (24.7, 32.0)
Hypertension, %	6719 (56.1)	10 208 (57)	3489 (60)
Systolic blood pressure, mm Hg	134 (124, 146)	134 (124, 145)	134 (125, 145)
Diastolic blood pressure, mm Hg	80 (75, 86)	80 (75, 87)	80 (76, 88)
Current smoker	1757 (14.7)	2820 (15.9)	1063 (18)
Family history of premature CAD	1527 (12.8)	2045 (11.5)	518 (9.0)
Glucose, mg/dL	95 (88, 102)	94 (88, 102)	93 (86, 101)
hsCRP, mg/L	4.10 (2.75, 6.70)	4.25 (2.85, 7.10)	4.70 (2.95, 8.05)
LDL cholesterol, mg/dL	109 (95, 119.5)	108 (94, 119)	106 (91, 118)
Non‐HDL cholesterol, mg/dL	135 (120, 147)	134 (118, 147)	132 (114, 146)
Apolipoprotein B, mg/dL	109 (97, 122)	109 (95, 122)	108 (93, 122)
Triglycerides, mg/dL	119 (87, 169)	118 (85, 169)	116 (83, 169)
HDL cholesterol, mg/dL	49 (41, 60)	49 (40, 60)	48 (40, 59)

CAD indicates coronary artery disease; hsCRP, high‐sensitivity C‐reactive protein; IQR, interquartile range; JUPITER, Justification for the Use of Statins in Prevention: an Intervention Trial; LDL‐c, low‐density lipoprotein cholesterol; LDL‐p, low density lipoprotein particle concentration; non‐HDL‐c, non‐high‐density lipoprotein cholesterol concentration; VLDL‐c, very low density lipoprotein cholesterol; VLDL‐p, very low density lipoprotein particle concentration.

**Figure 2:**
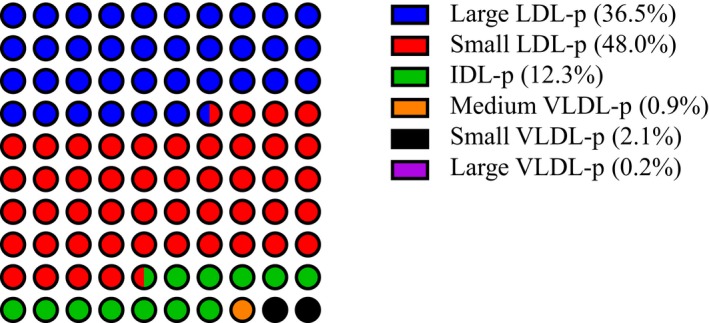
Schematic representation of the median proportion of atherogenic (LDL and VLDL) lipoprotein subclasses (median subclass particle concentration divided by median total LDL+VLDL particle concentration measured with NMR) at baseline in fasting samples in the rosuvastatin arm pre‐treatment. IDL‐p indicates intermediate density lipoprotein particle concentration; LDL‐p, low density lipoprotein particle concentration; NMR, nuclear magnetic resonance; VLDL‐p, very low density lipoprotein particle concentration.

Total LDL‐p and apoB were strongly correlated at baseline (Table 2), and LDL‐p was the most abundant atherogenic lipoprotein (Figure 2; Table 3). NMR‐measured VLDL/CM triglyceride was strongly correlated with chemically measured triglycerides and with large VLDL‐p (and progressively less with medium and small VLDL‐p). Calculated remnant cholesterol was (as expected based on the use of the Friedewald equation) perfectly correlated with triglyceride (*r*=1.00), moderately with apoB, non‐HDL‐c, and VLDL‐c (*r*=0.48, 0.60, and 0.58, respectively), and not with LDL‐c (*r*=0.01).

**Table 2:** Spearman Correlations Between Lipid and Lipoprotein Markers at Baseline (N=11 984)

	LDL‐c	Non‐HDL‐c	ApoB	Triglycerides	RC	HDL‐c	LDL Size	LDL‐p				VLDL Size	VLDL‐p				VLDLc	VLDL/CM Triglycerides
								Total	Large	Small	IDL		Total	Large	Medium	Small		
LDL‐c	···	0.75	0.51	0.01	0.01	0.05	0.15	0.40	0.33	0.09	0.19	−0.11	0.12	−0.01	0.06	0.13	0.58	0.05
Non‐HDL‐c	···	···	0.71	0.60	0.60	−0.27	−0.21	0.61	0.03	0.44	0.16	0.18	0.42	0.39	0.38	0.27	0.45	0.46
ApoB	···	···	···	0.48	0.48	−0.29	−0.21	0.79	0.13	0.53	0.18	0.16	0.39	0.36	0.37	0.23	0.42	0.43
Triglycerides	···	···	···	···	1.00	−0.49	−0.49	0.47	−0.32	0.59	0.04	0.42	0.52	0.64	0.53	0.28	0.58	0.69
RC	···	···	···	···	···	−0.49	−0.49	0.46	−0.32	0.59	0.41	0.42	0.52	0.63	0.53	0.28	0.58	
HDL‐c	···	···	···	···	···	···	0.57	−0.38	0.35	−0.60	0.16	−0.16	−0.38	−0.35	−0.38	−0.22	−0.40	−0.42
LDL size	···	···	···	···	···	···	···	−0.32	0.70	−0.77	0.10	−0.26	−0.39	−0.42	−0.42	−0.19	−0.43	−0.49
LDL‐p					···													
Total	···	···	···	···	···	···	···	···	0.17	0.71	0.09	0.18	0.29	0.31	0.34	0.13	0.33	0.37
Large	···	···	···	···	···	···	···	···	···	−0.45	−0.05	−0.38	−0.24	−0.41	−0.31	−0.05	−0.28	−0.39
Small	···	···	···	···	···	···	···	···	···	···	−0.14	0.33	0.41	0.50	0.47	0.18	0.48	0.55
IDL‐p	···	···	···	···	···	···	···	···	···	···	···	0.17	−0.03	0.09	0.14	−0.13	−0.04	0.07
VLDL size	···	···	···	···	···	···	···	···	···	···	···	···	0.03	0.76	0.35	−0.30	0.13	0.51
VLDL‐p					···													
Total	···	···	···	···	···	···	···	···	···	···	···	···	···	0.56	0.66	0.85	0.98	0.84
Large	···	···	···	···	···	···	···	···	···	···	···	···	···	···	0.58	0.24	0.70	0.87
Medium	···	···	···	···	···	···	···	···	···	···	···	···	···	···	···	0.22	0.66	0.79
Small	···	···	···	···	···	···	···	···	···	···	···	···	···	···	···	···	0.79	0.50
VLDL‐c	···	···	···	···	···	···	···	···	···	···	···	···	···	···	···	···	···	0.89
VLDL/CM triglycerides	···	···	···	···	···	···	···	···	···	···	···	···	···	···	···	···	···	···

All *P* values for Spearman correlations were <0.0001 except for the Spearman *r* of LDL‐c and triglycerides (*P*=0.175), LDL‐c and VLDL‐p_large_ (*P*=0.514), LDL‐c and RC (*P*=0.1813), non‐HDL‐c and LDL‐p_large_ (*P*=0.001), VLDL‐p_total_ and IDL‐p (*P*=0.0003), and VLDL‐p_total_ and VLDL size (*P*=0.0002). ApoB indicates apolipoprotein b; CM, chylomicrons; LDL‐c, low‐density lipoprotein cholesterol; LDL‐p, low‐density lipoprotein particle concentration; non‐HDL‐c, non‐high‐density lipoprotein cholesterol concentration; RC, remnant cholesterol; VLDL‐c, very low‐density lipoprotein cholesterol; VLDL‐p, very low‐density lipoprotein particle concentration.

**Table 3:** Baseline, Follow‐Up, and Change in Lipid and Lipoprotein Measures in the Placebo and Rosuvastatin Groups^a^

	Baseline	Year 1^b^	Absolute Change^c^	% Change
Lipids and apolipoproteins, mg/dL				
LDL‐c				
Placebo	110 (96, 119)	111 (96, 125)	3 (−8, 15)	2.7 (−7.4, 14.4)
Rosuvastatin	109 (96, 120)	55 (44, 71)	−51 (−65, −31)	−49.0 (−58.2, −32.8)
Non‐HDL‐c				
Placebo	135 (121, 147)	138 (121, 154)	3 (−9, 16)	2.4 (−6.4, 12.1)
Rosuvastatin	135 (121, 148)	77 (65, 96)	−56 (−71, −34)	−42.7 (−51.1, −27.7)
Apolipoprotein B				
Placebo	110 (97, 122)	106 (93, 119)	−3 (−13, 7)	−2.9 (−11.6, 6.3)
Rosuvastatin	110 (97, 123)	66 (57, 81)	−42 (−54, −27)	−39.4 (−47.6, −26.7)
Triglycerides				
Placebo	119 (88, 168)	120 (90, 167)	2 (−24, 25)	1.3 (−18.4, 24.7)
Rosuvastatin	120 (88, 171)	102 (77, 139)	−17 (−48, 5)	−15.5 (−32.8, 5.26)
NMR lipoproteins				
LDL size, nm diameter				
Placebo	21 (20.5, 21.4)	21 (20.5, 21.3)	0.0 (−0.3, 0.3)	0.0 (−1.5, 1.4)
Rosuvastatin	21 (20.5, 21.4)	20.6 (20.2, 21.0)	−0.3 (−0.8, 0.1)	−1.5 (−3.7, 0.5)
LDL‐p by subclass, nmol/L				
Total				
Placebo	1282 (1089, 1476)	1224 (1044, 1427)	−52 (−197, 95)	−4.2 (14.7, 8.3)
Rosuvastatin	1274 (1101, 1484)	771 (633, 964)	−487 (−674, −284)	−39.6 (−49.4, −24.7)
Large				
Placebo	457 (307, 602)	438 (276, 586)	−20 (−143, 104)	−5.3 (−31.0, 27.4)
Rosuvastatin	462 (304, 600)	174 (93, 306)	−237 (−391, −71)	−57.5 (−77, −24.4)
Small				
Placebo	612 (448, 841)	596 (440, 829)	−14 (−159, 127)	−2.6 (−22.9, 23.2)
Rosuvastatin	608 (446, 849)	494 (374, 624)	−127 (−320, 19)	−22.1 (−42.8, 4.5)
IDL‐p total, nmol/L				
Placebo	151 (96, 220)	139 (85, 201)	−14 (−82, 56)	−10.6 (−45.3, 49.0)
Rosuvastatin	156 (99, 225)	83 (52, 124)	−68 (−138, −4)	−45.9 (−69.4, −4.4)
VLDL size, nm diameter				
Placebo	49 (44.3, 53.7)	49.3 (44.6, 55.0)	0.6 (−3.7, 5.0)	1.3 (−7.3, 10.9)
Rosuvastatin	49 (44.3, 54.1)	50.2 (46.1, 55.1)	1.4 (−3.0, 5.9)	2.9 (−5.8, 12.7)
VLDL‐p by subclass, nmol/L				
Total				
Placebo	43.8 (30.1, 58.5)	44.1 (30.9, 60.8)	1.2 (−10.1, 13)	3.5 (−21.8, 35.4)
Rosuvastatin	43.3 (29.9, 58.8)	33.8 (23.5, 46.7)	−8.5 (−20.4, 3.2)	−19.6 (−40.5, 10.2)
Large				
Placebo	2.8 (1.3, 4.9)	3.0 (1.3, 5.6)	0.2 (−1.0, 1.6)	7.7 (−35.7, 75.9)
Rosuvastatin	2.8 (1.4, 5.0)	2.2 (1.1, 4.2)	−0.3 (−1.7, 0.7)	−15.4 (−50.0, 40.0)
Medium				
Placebo	11.8 (6.8, 18.7)	13.6 (7.6, 22.4)	1.9 (−3.2, 8.0)	16.7 (−26.7, 86.7)
Rosuvastatin	11.6 (6.8, 18.6)	10.9 (6.5, 17.1)	−0.6 (−6.0, 4.4)	−7.0 (−42.9, 52.0)
Small				
Placebo	27.1 (17.3, 38.1)	25 (16.3, 36.4)	−1.4 (−12.0, 9.3)	−6.0 (−37.8, 43.5)
Rosuvastatin	26.6 (17, 38.5)	19.4 (12.4, 28.0)	−6.9 (−17.3, 3.0)	−26.8 (−53.2, 16.4)
VLDL triglycerides, mg/dL				
Placebo	62.3 (42.7, 87.7)	64 (44.2, 94.1)	3.1 (−12.1, 20.2)	5.6 (−18.8, 35.8)
Rosuvastatin	62.3 (43.5, 88.3)	51.6 (36.5, 74.6)	−9.0 (−25.9, 5.5)	−15.2 (−35.8, 11.3)

Values obtained from individuals with both baseline and year 1 measurements (n=9222). HDL indicates high‐density lipoproteins; IDL, intermediate‐density lipoproteins; LDL‐c, low‐density lipoprotein cholesterol; LDL‐p, low‐density lipoprotein particle concentration; non‐HDL‐c, non‐high‐density lipoprotein cholesterol concentration; NMR, nuclear magnetic resonance; RC, remnant cholesterol; VLDL‐c, very low‐density lipoprotein cholesterol; VLDL‐p, very low‐density lipoprotein particle concentration.

a Median (25th percentile, 75th percentile).

b *P* values from the Wilcoxon signed‐rank test comparing baseline and year‐1 values were statistically significant (*P*<0.001) for all, with the exception of triglycerides among the placebo group (*P*=0.15).

c *P* values from the Wilcoxon rank‐sum test comparing the change among the rosuvastatin group with the change among the placebo group were <0.001 for all.

### Baseline Risk of Incident CVD

**Figure 3:**
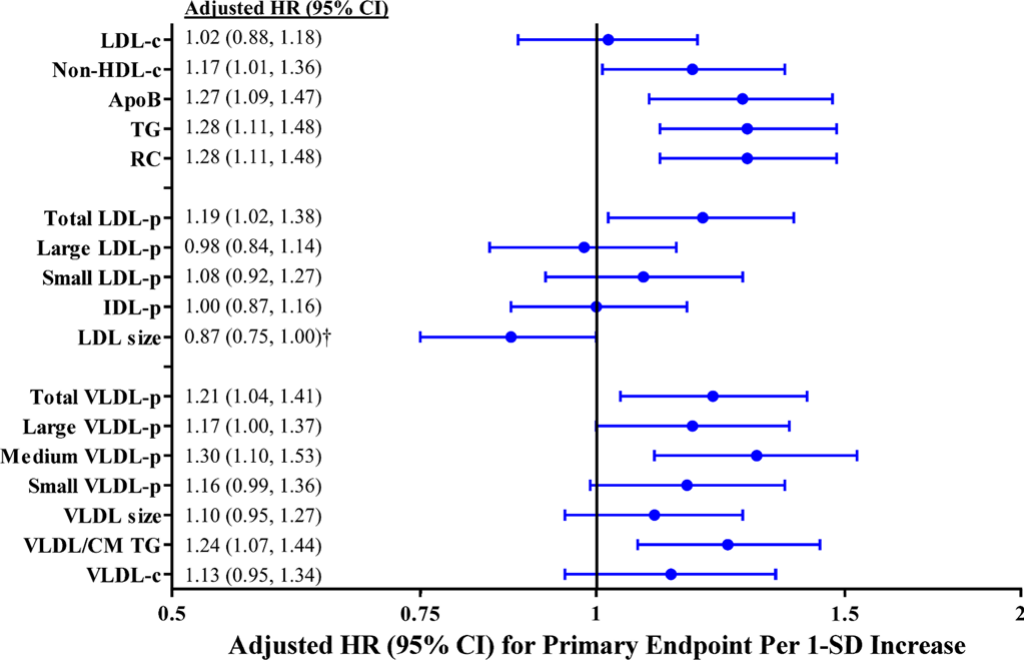
Adjusted* HR and 95%CI for the primary incident CVD end point in the JUPITER placebo group associated with baseline marker levels. ApoB indicates apolipoprotein B; CM, chylomicrons; HR, hazard ratio; IDL‐p, intermediate density lipoprotein particle concentration; JUPITER, Justification for the Use of Statins in Prevention: an Intervention Trial; LDL‐c, low‐density lipoprotein cholesterol; LDL‐p, low‐density lipoprotein particle concentration; non‐HDL‐c, non‐high‐density lipoprotein cholesterol concentration; RC, calculated remnant cholesterol; TG, triglycerides; VLDL‐c, very low‐density lipoprotein cholesterol; VLDL‐p, very low‐density lipoprotein particle concentration. *Adjusted for age, sex, race, smoking status, family history of premature coronary disease, body‐mass index, systolic blood pressure, fasting glucose, and hsCRP. ^†^ LDL size was no longer associated with either end point after adjusting for LDL‐p and HDL‐c. The following biomarkers were natural‐log‐transformed: triglyceride, RC, small LDL‐p, IDL‐p, all VLDL‐p subclasses, VLDL/CM triglycerides, and VLDL‐c.

Among the 11 984 JUPITER participants, a total of 296 primary events and 489 expanded end points occurred over a median follow‐up of 2.0 (maximum 5.0) years, representing 27 864 person‐years. Among participants in the placebo‐allocated arm (N=6050; 189 events; Figure 3), LDL‐c was not associated with increased risk of the primary end point, but significant associations were observed for non‐HDL‐c (1.17 [1.01, 1.36]), triglycerides (1.28 [1.10, 1.48]), and apoB (1.27 [1.09, 1.47]). For the expanded end point that included all‐cause mortality (291 events; Table 4), associations were generally attenuated and became nonsignificant for non‐HDL‐c and apoB. Tertile analysis demonstrated a graded increase in risk with increasing levels of these markers but not LDL‐c (Table 5).

**Table 4:** Adjusted HR and 95%CI for the Expanded End Point (Incident CVD and All‐Cause Mortality) in the Placebo Group at Baseline and the Rosuvastatin Group On‐Treatment

	Baseline Risk (Placebo; N=6050)		Residual Risk (Rosuvatatin; N=4386)	
	CVD and All‐Cause Death		CVD and All‐Cause Death	
	HR per 1 SD Higher^a^ (95%CI)	*P* Value	HR per 1 SD Higher^a^, ^b^ (95%CI)	*P* Value
Lipids and apolipoproteins (SD), mg/dL				
LDL‐c	0.90 (0.80, 1.00)	0.061	1.14 (1.02, 1.28)	0.024
Non‐HDL‐c	1.00 (0.88, 1.12)	0.979	1.16 (1.02, 1.32)	0.023
Apolipoprotein B	1.06 (0.94, 1.19)	0.352	1.19 (1.01, 1.40)	0.033
Triglycerides^c^	1.18 (1.05, 1.33)	0.005	1.10 (0.89, 1.36)	0.391
RC^c^	1.19 (1.05, 1.33)	0.005	1.10 (0.89, 1.37)	0.390
NMR lipoproteins (SD)				
LDL size	0.85 (0.75, 0.95)	0.006	1.21 (0.98, 1.50)	0.081
LDL‐p				
Total	1.01 (0.89, 1.14)	0.923	1.07 (0.88, 1.31)	0.504
Large	0.93 (0.82, 1.05)	0.218	1.18 (0.95, 1.47)	0.144
Small^c^	0.97 (0.86, 1.08)	0.518	0.87 (0.72, 1.05)	0.153
IDL‐p^c^	0.98 (0.87, 1.10)	0.746	0.98 (0.83, 1.15)	0.760
VLDL size	1.08 (0.96, 1.21)	0.206	0.72 (0.57, 0.91)	0.005
VLDL‐p				
Total^c^	1.25 (1.11, 1.41)	0.0003	1.32 (1.08, 1.61)	0.006
Large^c^	1.19 (1.05, 1.35)	0.001	1.03 (0.85, 1.26)	0.762
Medium^c^	1.22 (1.08, 1.39)	0.002	1.04 (0.84, 1.27)	0.741
Small^c^	1.16 (1.04, 1.29)	0.007	1.56 (1.25, 1.95)	<0.0001
VLDL/chylomicron triglycerides c	1.27 (1.13, 1.42)	<0.0001	1.10 (0.91, 1.34)	0.325
VLDL‐c^c^	1.10 (0.95, 1.27)	0.211	1.27 (1.05, 1.54)	0.014

CHD indicates coronary heart disease; CVD, cardiovascular disease; HDL, high‐density lipoprotein; hsCRP, high‐sensitivity C‐reactive protein; IDL, intermediate‐density lipoprotein; LDL‐c, low‐density lipoprotein cholesterol; LDL‐p, low density lipoprotein particle concentration; NMR, nuclear magnetic resonance; non‐HDL‐c, non‐high density lipoprotein cholesterol concentration; RC, remnant cholesterol; VLDL‐c, very low density lipoprotein cholesterol; VLDL‐p, very low density lipoprotein particle concentration.

a Adjusted for age, race, sex, race, family history of CHD, smoking, systolic blood pressure, fasting glucose, body mass index, and the natural logarithm of hsCRP.

b Baseline SDs used to allow for comparison.

c Variable was log‐transformed (ln), and risk is per change in SD of the natural log of the variable.

**Table 5:** Baseline Lipid and Lipoproteins by Tertile in Relation to End Points in the Placebo Arm

	Adjusted HR (95%CI)^a^			
	Lowest Tertile	Middle Tertile	Highest Tertile	*P*_trend_ b
Lipids and apolipoproteins				
LDL‐c				
Range, mg/dL	≤100	100 to 116	>116	
Primary end point	Ref	0.99 (0.71, 1.14)	1.08 (0.76, 1.55)	0.66
Secondary end point	Ref	0.77 (0.59, 1.00)	0.85 (0.64, 1.12)	0.22
Non‐HDL‐c				
Range, mg/dL	≤125	125 to 143	>143	
Primary end point	Ref	1.18 (0.82, 1.69)	1.33 (0.93, 1.92)	0.122
Secondary end point	Ref	0.99 (0.75, 1.31)	1.06 (0.80, 1.41)	0.700
Apolipoprotein B				
Range, mg/dL	≤101	101 to 117	>117	
Primary end point	Ref	1.42 (1.00, 2.05)	1.54 (1.06, 2.22)	0.024
Secondary end point	Ref	1.09 (0.83, 1.44)	1.06 (0.79, 1.41)	0.701
Triglycerides				
Range, mg/dL	≤96	96 to 147	>147	
Primary end point	Ref	1.59 (1.08, 2.33)	1.84 (1.26, 2.69)	0.002
Secondary end point	Ref	1.40 (1.03, 1.88)	1.57 (1.16, 2.11)	0.003
NMR lipoproteins				
LDL size				
Range, nm	≤20.7	20.7 to 21.2	>21.2	
Primary end point	Ref	0.67 (0.47, 0.97)	0.77 (0.54, 1.09)	0.131
Secondary end point	Ref	0.67 (0.50, 0.89)	0.72 (0.54, 0.95)	0.019
LDL‐p				
Total				
Range, nmol/L	≤1147	1147 to 1390	>1390	
Primary end point	Ref	1.38 (0.95, 2.00)	1.57 (1.09, 2.28)	0.017
Secondary end point	Ref	1.12 (0.85, 1.48)	1.03 (0.77, 1.38)	0.828
Large				
Range	≤364	364 to 557	>557	
Primary end point	Ref	0.75 (0.52, 1.09)	1.03 (0.73, 1.45)	0.849
Secondary end point	Ref	0.76 (0.57, 1.01)	0.90 (0.68, 1.19)	0.466
Small				
Range, nmol/L	≤485	485 to 728	>728	
Primary end point	Ref	1.29 (0.89, 1.88)	1.44 (0.99, 2.10)	0.059
secondary end point	Ref	1.13 (0.85, 1.51)	1.14 (0.84, 1.53)	0.402
IDL‐p				
Range	≤115	115 to 193	>193	
Primary end point	Ref	1.15 (0.82, 1.63)	0.99 (0.69, 1.42)	0.967
Secondary end point	Ref	1.08 (0.82, 1.42)	1.02 (0.76, 1.36)	0.893
VLDL size				
Range, nm	≤45.6	45.6 to 51.7	>51.7	
Primary end point	Ref	1.23 (0.86, 1.77)	1.30 (0.90, 1.88)	0.164
Secondary end point	Ref	1.31 (0.99, 1.75)	1.26 (0.93, 1.69)	0.128
VLDL‐p				
Total				
Range, nmol/L	≤34.6	34.6 to 53.2	>53.2	
Primary end point	Ref	1.14 (0.77, 1.66)	1.55 (1.08, 2.21)	0.014
Secondary end point	Ref	1.21 (0.90, 1.64)	1.54 (1.15, 2.05)	0.003
Large				
Range, nmol/L	≤1.7	1.7 to 4.0	>4.0	
Primary end point	Ref	1.45 (1.01, 2.07)	1.26 (0.86, 1.84)	0.246
Secondary end point	Ref	1.44 (1.08, 1.93)	1.41 (1.04, 1.90)	0.028
Medium				
Range, nmol/L	≤8.1	8.1 to 15.7	>15.7	
Primary end point	Ref	1.36 (0.92, 2.00)	1.79 (1.24, 2.60)	0.002
Secondary end point	Ref	1.18 (0.87, 1.60)	1.72 (1.28, 2.29)	0.0002
Small				
Range, nmol/L	≤20.9	20.9 to 34.3	>34.3	
Primary end point	Ref	1.12 (0.77, 1.62)	1.31 (0.91, 1.86)	0.139
Secondary end point	Ref	1.31 (0.97, 1.78)	1.45 (1.08, 1.95)	0.014
VLDL triglycerides				
Range, mg/dL	≤49.1	49.1 to 77.5	>77.5	
Primary end point	Ref	1.30 (0.89, 1.89)	1.49 (1.03, 2.16)	0.035
Secondary end point	Ref	1.29 (0.96, 1.75)	1.59 (1.18, 2.13)	0.002

Tertiles derived from the baseline population. HR indicates hazard ratio; hsCRP, high‐sensitivity C‐reactive protein; IDL‐p, intermediate‐density particle concentration; LDL‐c, low‐density lipoprotein cholesterol; LDL‐p, low‐density lipoprotein particle concentration; non‐HDL‐c, non‐high‐density lipoprotein cholesterol concentration; RC, remnant cholesterol; Ref, reference value; VLDL‐c, very low‐density lipoprotein cholesterol; VLDL‐p, very low‐density lipoprotein particle concentration.

a Adjusted for adjusted for age, sex, race, smoking status, family history of premature coronary disease, body‐mass index, systolic blood pressure, fasting glucose, and hsCRP.

b *P* for nonlinear trend.

For NMR‐measured lipoproteins, a significant association was noted for LDL‐p and the primary end point (1.19 [1.02, 1.38]) but not for the individual LDL subclasses. Smaller LDL size was a marker of increased risk, but this was no longer significant after additionally adjusting for LDL‐p—as previously suggested^16^—and HDL‐c.

Conversely, most VLDL‐p subfractions, as well as total VLDL‐p and NMR‐determined VLDL/CM triglycerides, were associated with increased risk of the primary (Figure 3) or expanded (Table 4) end point. The magnitude of this risk was similar to that of chemically measured triglycerides. Because VLDL‐p subfractions were comparably associated with increased risk, there was no independent association for average VLDL particle size. Total NMR‐measured VLDL‐c was not significantly associated with the primary or expanded end points at baseline. Overall, when the baseline analysis was performed in the entire cohort adjusting for known randomized statin allocation as a covariable, the results were similar.

### Lipoprotein Subclass Response to Statin Therapy

**Figure 4:**
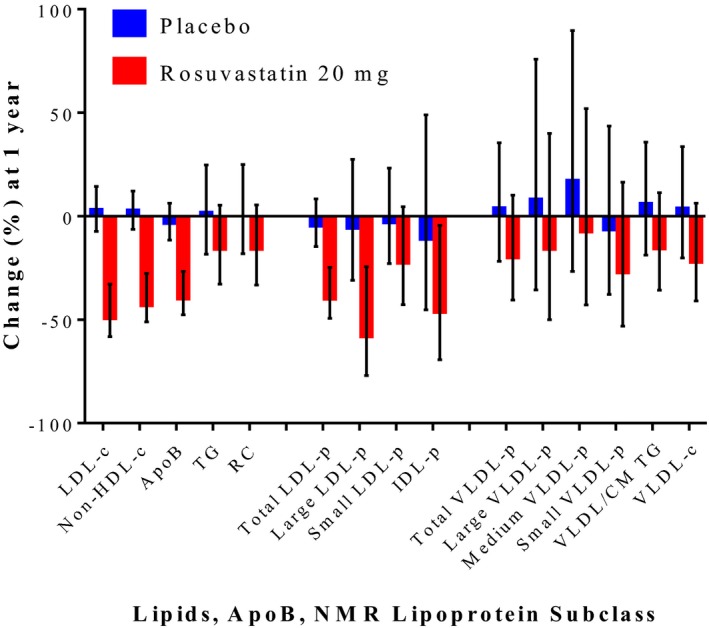
Median percentage change in lipids, apoB, and NMR‐measured lipoprotein subclasses in the placebo and statin‐treated arms in JUPITER. Error bars represent 25th and 75th percentiles. CM indicates chylomicrons; IDL‐p, intermediate‐density lipoprotein particle concentration; JUPITER, Justification for the Use of Statins in Prevention: an Intervention Trial; LDL‐c, low‐density lipoprotein cholesterol; LDL‐p, low‐density lipoprotein particle concentration; NMR, nuclear magnetic resonance; non‐HDL‐c, non‐high‐density lipoprotein cholesterol concentration; RC, calculated remnant cholesterol; TG, triglycerides; VLDL‐c, very low‐density lipoprotein cholesterol; VLDL‐p, very low density lipoprotein particle concentration.

Rosuvastatin therapy produced large median (25th, 75th percentile) percentage reductions in LDL‐c (−49.0 [−58.2, −32.8]%), apoB (−39.4 [−47.6, −26.7]%), and non‐HDL‐c (−42.7 [−51.1, −27.7]%; Figure 4; Table 3) and resulted in greater reductions in large (−57.5 [−77.0, −24.4]%) versus small (−22.1 [−42.8, 4.5]%) LDL‐p. Triglycerides and VLDL‐p subfractions showed smaller, more variable responses to statin therapy, and many participants had no reduction after a year of statin therapy.

### Residual Risk of CVD

**Figure 5:**
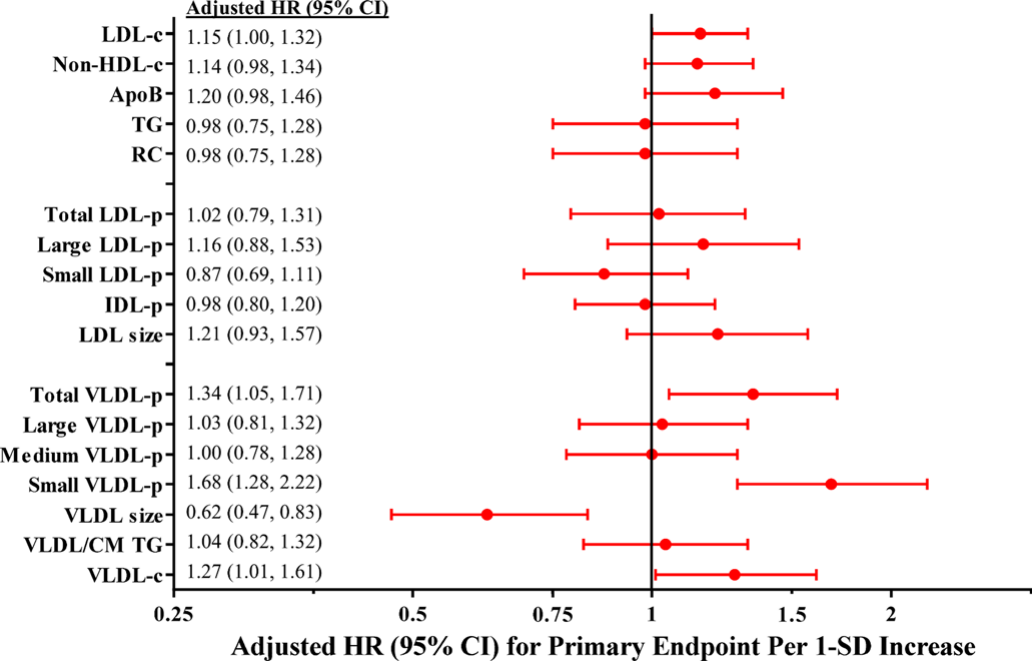
On‐statin adjusted* HR and 95%CI for the primary incident CVD end point in the JUPITER rosuvastatin 20‐mg group. CM indicates chylomicrons; HR, hazard ratio; IDL‐p, intermediate‐density lipoprotein particle concentration; JUPITER, Justification for the Use of Statins in Prevention: an Intervention Trial; LDL‐c, low‐density lipoprotein cholesterol; LDL‐p, low‐density lipoprotein particle concentration; non‐HDL‐c, non‐high‐density lipoprotein cholesterol concentration; RC, calculated remnant cholesterol; SD, standard deviation; TG, triglycerides; VLDL‐c, very low‐density lipoprotein cholesterol; VLDL‐p, very low‐density lipoprotein particle concentration. *Adjusted for age, sex, race, smoking status, family history of premature coronary disease, body‐mass index, systolic blood pressure, fasting glucose, and hsCRP. The following biomarkers were natural‐log‐transformed: triglyceride, RC, small LDL‐p, IDL‐p, all VLDL‐p subclasses, VLDL/CM triglycerides, and VLDL‐c.

Among participants in the statin‐allocated arm with on‐statin measures (N=4386), median LDL‐c was 55 mg/dL, and triglyceride was 102 mg/dL. In this population, standard lipid and apolipoprotein measures were marginally associated with risk (73 primary end‐point events and 108 expanded end‐point events; Figure 5; Tables 4 and 6), including apoB, non‐HDL‐c, and LDL‐c.

**Table 6:** On‐Treatment Lipid and Lipoproteins by Tertile in Relation to End Points in the Rosuvastatin Arm

	Adjusted HR (95%CI)^a^			
	Lowest Tertile	Middle Tertile	Highest Tertile	*P*_trend_ b
Lipids and apolipoproteins				
LDL‐c				
Range, mg/dL	≤47	47 to 63	>63	
Primary end point	Ref	0.74 (0.39, 1.42)	1.73 (1.00, 2.98)	0.034
Secondary end point	Ref	0.69 (0.41, 1.17)	1.41 (0.91, 2.21)	0.099
Non‐HDL‐c				
Range, mg/dL	≤68	68 to 87	>87	
Primary end point	Ref	0.80 (0.43, 1.49)	1.53 (0.89, 2.63)	0.111
Secondary end point	Ref	0.81 (0.48, 1.34)	1.60 (1.02, 2.50)	0.030
Apolipoprotein B				
Range, mg/dL	≤60	60 to 75	>75	
Primary end point	Ref	0.99 (0.54, 1.80)	1.47 (0.83, 2.58)	0.169
Secondary end point	Ref	0.95 (0.57, 1.58)	1.55 (0.98, 2.47)	0.049
Triglycerides				
Range, mg/dL	≤85	85 to 122	>122	
Primary end point	Ref	0.98 (0.55, 1.74)	1.04 (0.59, 1.85)	0.883
Secondary end point	Ref	1.05 (0.65, 1.68)	1.17 (0.73, 1.88)	0.506
NMR lipoproteins				
LDL size				
Range, nm	≤20.3	20.3 to 20.8	>20.8	
Primary end point	Ref	0.75 (0.40, 1.40)	1.58 (0.91, 2.72)	0.100
Secondary end point	Ref	0.74 (0.44, 1.25)	1.48 (0.94, 2.32)	0.078
LDL‐p				
Total				
Range, nmol/L	≤678	678 to 879	>879	
Primary end point	Ref	0.96 (0.54, 1.71)	1.09 (0.61, 1.92)	0.772
Secondary end point	Ref	0.74 (0.45, 1.22)	1.14 (0.73, 1.79)	0.528
Large				
Range	≤117	117 to 246	>246	
Primary end point	Ref	1.34 (0.75, 2.39)	1.38 (0.76, 2.50)	0.296
Secondary end point	Ref	1.03 (0.64, 1.68)	1.28 (0.80, 2.06)	0.291
Small				
Range, nmol/L	≤415	415 to 572	>572	
Primary end point	Ref	0.69 (0.40, 1.22)	0.63 (0.36, 1.13)	0.117
Secondary end point	Ref	0.69 (0.43, 1.11)	0.81 (0.51, 1.28)	0.361
IDL‐p				
Range	≤62	62 to 106	>106	
Primary end point	Ref	0.80 (0.48, 1.36)	0.49 (0.27, 0.91)	0.024
Secondary end point	Ref	0.79 (0.50, 1.23)	0.67 (0.42, 1.07)	0.090
VLDL size				
Range, nm	≤47.4	47.4 to 53.1	>53.1	
Primary end point	Ref	0.83 (0.50, 1.38)	0.31 (0.15, 0.63)	0.002
Secondary end point	Ref	0.81 (0.53, 1.23)	0.40 (0.23, 0.70)	0.001
VLDL‐p				
Total				
Range, nmol/L	≤27.0	27.0 to 41.3	>41.3	
Primary end point	Ref	1.40 (0.75, 2.61)	1.67 (0.92, 3.04)	0.095
Secondary end point	Ref	1.29 (0.78, 2.14)	1.59 (0.98, 2.58)	0.059
Large				
Range, nmol/L	≤1.4	1.4 to 3.3	>3.3	
Primary end point	Ref	1.94 (1.11, 3.38)	1.01 (0.53, 1.91)	0.929
Secondary end point	Ref	1.63 (1.04, 2.56)	0.94 (0.56, 1.56)	0.898
Medium				
Range, nmol/L	≤7.9	7.9 to 14.4	>14.4	
Primary end point	Ref	0.84 (0.48, 1.49)	0.85 (0.49, 1.50)	0.582
Secondary end point	Ref	0.97 (0.61, 1.55)	0.96 (0.60, 1.54)	0.865
Small				
Range, nmol/L	≤14.5	14.5 to 24.3	>24.3	
Primary end point	Ref	2.29 (1.09, 4.82)	3.73 (1.86, 7.49)	<0.0001
Secondary end point	Ref	1.97 (1.13, 3.43)	2.56 (1.51, 4.34)	0.0005
VLDL triglycerides				
Range, mg/dL	≤41.1	41.1 to 64.4	>64.4	
primary end point	Ref	1.32 (0.74, 2.35)	1.17 (0.64, 2.12)	0.629
Secondary end point	Ref	1.16 (0.72, 1.87)	1.24 (0.77, 2.01)	0.375

Tertiles derived from on‐treatment (rosuvastatin) group at 12 months. HR indicates hazard ratio; hsCRP, high‐sensitivity C‐reactive protein; IDL‐p, intermediate‐density particle concentration; LDL‐c, low‐density lipoprotein cholesterol; LDL‐p, low‐density lipoprotein particle concentration; non‐HDL‐c, non‐high‐density lipoprotein cholesterol concentration; RC, remnant cholesterol; Ref, reference value; VLDL‐c, very low density lipoprotein cholesterol; VLDL‐p, very low density lipoprotein particle concentration.

a Adjusted for adjusted for age, sex, race, smoking status, family history of premature coronary disease, body‐mass index, systolic blood pressure, fasting glucose, and hsCRP.

b *P* for nonlinear trend.

On statin therapy, each SD increment in total VLDL‐p, driven by the smallest VLDL‐p subclass, was associated with residual risk (Figure 5). Adjusted hazard ratios (95%CIs) per increasing tertile of on‐treatment small VLDL‐p were 1.00 (Ref.), 2.29 (1.09, 4.82), and 3.73 (1.86, 7.49), *P* for trend <0.0001 (Table 6). For each SD greater average VLDL size, reflecting a shift away from small VLDL‐p, there was a 40% relative risk reduction. In sensitivity analyses these associations remained significant after adjustment for other NMR lipoproteins (1.83 [1.35, 2.50]) and incrementally for HDL‐c (1.77 [1.29, 2.43]) or HDL‐p (1.78 [1.31, 2.43]). On‐treatment NMR‐measured VLDL‐c was associated with both the primary and expanded end points. This risk was driven by the cholesterol contained in the small VLDL subclass (primary end point 1.74 [1.32, 2.30]; expanded end point 1.59 [1.27, 1.98]). In contrast, no associations were found for on‐treatment triglycerides, NMR‐measured VLDL/CM triglycerides, formulaic remnant cholesterol, or large and medium VLDL‐p subfractions.

### Secondary Cohort Examination

Participants in CATHGEN with LDL‐c <130 mg/dL and triglycerides <500 mg/dL were demographically similar to JUPITER participants in age (mean [SD] 60.7±11.7 years), sex (62.3% male), and racial (74.2% white) distribution as well as based on body‐mass index (30.1±7.4 kg/m^2^) and systolic blood pressure (144±25 mm Hg). Among these 2149 individuals, 154 events occurred over median (maximum) 5.0 (13.3) years of follow‐up. Total VLDL‐p was significantly associated with risk of myocardial infarction, driven by the small VLDL lipoprotein subclass (Table 7). As with larger VLDL‐p subclasses, VLDL/CM triglyceride concentration was not significantly associated with risk. Although restriction to those confirmed as having a prescribed statin reduced sample size to only 833 individuals, the pattern of the associations was unchanged, but the confidence intervals in this much smaller subgroup were wider and no longer significant. When no LDL‐c or statin‐based selection criteria were applied, risk of myocardial infarction among all comers with NMR measurements in the cohort was not significantly associated with VLDL‐p levels. Similarly, the magnitude of risk associated with LDL‐p became larger as the cohort was progressively more selected based on LDL‐c <130 mg/dL and statin prescription status.

**Table 7:** Adjusted HR (95%CI) for Myocardial Infarction Among Participants in the CATHGEN Registry (Replication Cohort), Among All Comers, Those With LDL‐c <130 mg/dL and Triglyceride <500 mg/dL, as Well as Those With Confirmed Statin Prescription at Time of NMR Measurement

	CATHGEN Registry: Adjusted HR^a^ (95%CI) of Myocardial Infarction Per‐1 SD Incremental Increase in Lipoprotein		
	All Participants (n=4721; 346 Cases)	LDL‐c <130 mg/dL TG <500 mg/dL (n=2149; 154 Events)	Statin Prescription (n=833; 59 Events)
LDL‐p			
Total	1.17 (1.05, 1.31)	1.28 (1.07, 1.52)	1.61 (1.20, 2.14)
Large	1.02 (0.91, 1.14)	0.92 (0.79, 1.08)	1.33 (0.99, 1.80)
Small^b^	1.12 (0.99, 1.26)	1.21 (0.99, 1.49)	1.54 (1.04, 2.29)
IDL‐p^b^	0.97 (0.87, 1.07)	0.95 (0.81, 1.12)	0.93 (0.71, 1.21)
VLDL‐p			
Total^b^	1.11 (0.98, 1.26)	1.23 (1.01, 1.51)	1.19 (0.86, 1.63)
Large^b^	0.96 (0.86, 1.07)	1.01 (0.86, 1.19)	1.04 (0.80, 1.36)
Medium^b^	1.01 (0.90, 1.13)	1.03 (0.87, 1.22)	1.11 (0.83, 1.48)
Small^b^	1.10 (0.97, 1.24)	1.27 (1.02, 1.57)	1.35 (0.96, 1.90)
TG (VLDL/CM)^b^	1.06 (0.94, 1.18)	1.12 (0.94, 1.32)	1.10 (0.83, 1.46)

CHD indicates coronary heart disease; CM, chylomicrons; IDL‐p, intermediate‐density lipoprotein particle concentration; LDL‐p, low‐density lipoprotein particle concentration; RC, remnant cholesterol; TG, triglycerides; VLDL‐p, very low‐density lipoprotein particle concentration.

a Adjusted for age, race, sex, family history of CHD, smoking, systolic blood pressure, fasting glucose, and body mass index.

b Variable was log‐transformed (ln), and risk is per change in SD of the natural log of the variable.

### Prediction Model Performance

In JUPITER the C‐statistic (a measure of model discrimination) for the baseline CVD risk model (built with 12 clinical and standard lipid measure variables; 12 degrees of freedom) for the placebo group was not significantly changed (*P*=0.50) with the addition of all NMR lipoprotein measures (24 variables): 0.664 (95%CI=0.618, 0.710) to 0.668 (0.623, 0.714). A baseline parsimonious model (which statistically selected among the 24 candidate variables those that most significantly contributed to CVD prediction) identified age, sex, smoking, family history, and medium VLDL‐p as top predictor variables. Conversely for residual risk models, discrimination was significantly improved by a model that included all of the NMR variables (24 degrees of freedom) compared to a model with only clinical and standard lipid variables (12 degrees of freedom): C=0.780 (0.718, 0.842) versus 0.712 (0.636, 0.788); *P*=0.0001. The parsimonious model for residual risk selected age, sex, smoking, hsCRP, VLDL‐c, small LDL‐p, and VLDL size as top predictors.

## Discussion

This study was designed to identify circulating lipoproteins measured by ^1^H NMR associated with CVD risk among individuals with naturally or pharmacologically low (<130 mg/dL) LDL‐c. We observed that, among such individuals, risk was associated with lipoprotein particle concentration, including LDL and VLDL subfractions. Intriguingly, among individuals on statin therapy (median LDL‐c 55 mg/dL), the lipoprotein subclass small VLDL‐p (and its associated cholesterol) conferred an ≈70% per‐SD increase in residual risk, corresponding to a 3.7‐fold increase in risk among those in the highest tertile. These observations were extended using a distinct, diverse cohort referred for cardiac catheterization (CATHGEN); as refinement of the population based on LDL‐c <130 mg/dL or statin prescription was undertaken, the magnitude of risk related to lipoprotein subclasses appeared amplified (including small VLDL‐p). Exploratory analyses suggested that on‐statin residual risk prediction could be significantly improved beyond known clinical and standard lipid risk factors with the inclusion of NMR‐based VLDL measurements. Overall, our results suggest a potential role for VLDL lipoproteins in the development of CVD events, particularly among individuals with lower LDL‐c—a rapidly growing population in clinical practice^1^, ^2^ in whom event rates nonetheless remain unacceptably high.^3^, ^17^

We observed that baseline apoB and LDL‐p were associated with risk of incident CVD. Although both measures were highly correlated (*r*=0.79), the magnitude of risk associated with apoB was somewhat greater than that for LDL‐p, consistent with findings from the Women's Health Study.^14^ In addition to LDL, apoB is also present on VLDL and lipoprotein(a), the latter of which also carried increased risk in JUPITER.^18^

Triglycerides and VLDL/CM measures were associated with risk in the JUPITER placebo group, whereas the small VLDL‐p subclass (but not triglycerides) was strongly associated with risk in the statin group. VLDL (also referred to as triglyceride‐rich lipoproteins) are believed to confer atherogenic risk related to their cholesterol content (ie, remnant cholesterol),^19^ which can be deposited directly into the arterial wall without modification.^20^ Why the magnitude of risk related to VLDL‐p appeared to differ based on statin allocation is unclear and deserves further investigation. It is possible that statin‐related suppression of inflammation could modify the relationship between VLDL and atherosclerosis.^19^

Our study is novel in its consideration of NMR‐based atherogenic lipoprotein measures among dedicated populations of individuals with low (<130 mg/dL) LDL‐c, an increasingly prevalent population in the era of LDL‐c reduction. Our findings support and extend previous observations from other cohorts, wherein lipoprotein particle concentration measured by apoB and NMR (LDL‐p) was associated with CVD events among statin‐treated secondary prevention individuals.^21^ Additionally, no other study has assessed the significance of on‐statin levels of NMR‐measured VLDL lipoproteins in relation to residual CVD risk. Our results help frame those of previous studies, wherein on‐statin triglyceride levels have been inconsistently associated with residual risk,^22^ suggesting the need for more detailed VLDL phenotyping. Our results also support and extend recent efforts to more comprehensively profile statin effects on the spectrum of the lipid and lipoprotein milieu.^23^

The risk estimates observed with NMR are largely in agreement with those recently obtained in the same cohort using electrospray ion mobility,^24^ except that stronger association was noted for NMR‐measured small VLDL‐p with residual risk. In JUPITER, measurements of small VLDL by NMR and ion mobility were only modestly correlated (Spearman *r*=0.22). In other cohorts, NMR and ion mobility have also demonstrated differential resolution of the proatherogenic effects of VLDL.^25^ The basis for such differences may be due to methodological differences, with NMR providing better measurement of lipid‐rich lipoprotein particles such as VLDL, while ion mobility may better capture protein‐rich particles.

Our study has several potential limitations. First, the follow‐up duration in JUPITER was relatively short for a primary prevention study (due to early termination of the trial for benefit by the Data Safety and Monitoring Board). Nonetheless, event rates were sufficiently high to detect a number of expected and novel associations, and the longer follow‐up in CATHGEN (median [maximum] 5.0 [13.3] years) suggested longitudinal extrapolation of these results. Second, all JUPITER study participants had elevated hsCRP. Systemic inflammation has been shown to affect the composition of the lipoprotein milieu, including VLDL.^26^ At a minimum, it has been estimated that more than 1 in 50 adult Americans would meet the JUPITER inclusion criteria.^27^ Furthermore, hsCRP was not part of the selection criteria in CATHGEN, suggesting that these findings may be more broadly generalizable. Finally, multiple comparisons were performed, increasing the chance of a type I error. However, examination of an independent cohort supported the key associations observed, and the findings are supported by prior biologic and epidemiological studies. Nonetheless, given the multiplicity of hypotheses tested, these results should be viewed as hypothesis‐generating and require further validation in additional cohorts.

## Conclusions

In conclusion, among individuals with low (<130 mg/dL) LDL‐c, risk remains associated with lipoprotein particle concentration, including VLDL lipoproteins. We observed on‐statin risk strongly associated with small VLDL‐p—risk that appears to be triglyceride‐independent and instead related to the cholesterol carried by these small VLDL particles. Overall, these hypothesis‐generating findings draw attention to the potential importance of VLDL lipoproteins, in particular small remnant particles, which are associated with risk but are currently not the target of prediction or therapeutic intervention. Additional studies are needed to validate these findings and assess the potential causal role of these lipoproteins as determinants of CVD risk.

## Sources of Funding

Lawler received support from NIH T32 (HL007575), NIH Loan Repayment Program, and Brigham and Women's Hospital, and currently receives support from the Peter Munk Cardiac Centre, University Health Network, the University of Toronto, and the Heart and Stroke/Richard Lewar Center for Excellence in Cardiovascular Research. Akinkoulie receives support from NIH T32 (HL007575). The research for this article was supported by the National Heart, Lung, and Blood Institute of the NIH under Award Numbers R01HL117861 and R01HL117861‐S1 and ‐S2 to Mora, and by the Molino Family Trust. The content is solely the responsibility of the authors and does not necessarily represent the official views of the NIH. The funding agency had no role in the design and execution of the current study. The JUPITER trial (NCT00239681) was funded by AstraZeneca, who had no role in the design and execution of this study. LipoScience (now LabCorp) performed the NMR lipoprotein analysis for this study at no additional cost.

## Disclosures

Mora received research grant support from Atherotech Diagnostics for research outside the current work and served as a consultant to Amgen, Lilly, Pfizer, Cerenis Therapeutics, and Quest Diagnostics. Glynn received research grant support from AstraZeneca. Ridker received research grant support from AstraZeneca, Kowa, Novartis, Amgen, Pfizer, and NHLBI and is listed as a coinventor on patents held by the Brigham and Women's Hospital related to the use of inflammatory biomarkers in CVD (licensed to AstraZeneca and Siemens). All other authors report no disclosures. Shah, Craig, and Kraus received research funding from LipoScience (now LabCorp) during this funding period. Chu is currently an employee at Merck but was not during the conduct of this study.
